# A qualitative exploration of service users' experiences of weight management conversations in a mental health setting

**DOI:** 10.1016/j.pecinn.2025.100389

**Published:** 2025-03-22

**Authors:** Emma Kemp, Catherine Haighton, Sally Faulkner, Kate McBride, Maria Raisa Jessica Aquino, Rob Wilson, Milica Vasiljevic, Craig Robson, Mish Loraine, Jill Harland, Angela M. Rodrigues

**Affiliations:** a School of Psychology, University of Sheffield, Sheffield, S10 2TN, UK; b Department of Social Work, Education and Community Wellbeing, Northumbria University, Newcastle upon Tyne NE7 7XA, UK; c Cumbria, Northumberland, Tyne & Wear NHS Foundation Trust, St Nicholas Hospital, Newcastle upon Tyne NE3 3XT, UK; d Population Health Sciences Institute, Newcastle University, Newcastle upon Tyne, NE1 4AX, UK; e Department of Sociology, Manchester Metropolitan University, Manchester, M15 6BX, UK; f Department of Psychology, Durham University, Durham DH1 3LE, UK; g Northumbria Healthcare NHS Foundation Trust, North Tyneside General Hospital, NE29 8NH, UK; h Holy Jesus Hospital, City Road, Newcastle upon Tyne NE1 2AS, UK; i Department of Psychology, Northumbria University, Newcastle upon Tyne NE1 8ST, UK

**Keywords:** Weight management conversations, Serious mental illness, Making every contact count (MECC), Service user experiences

## Abstract

**Objective:**

Healthcare professionals often use opportunistic weight management conversations, aligned with the Making Every Contact Count (MECC) approach, to provide motivational support to service users. While research supports this practice from the professionals' perspective, the views of service users on these interactions remain understudied. The aim of this study was to explore the experiences of service users with serious mental illness regarding weight management conversations with healthcare professionals.

**Methods:**

Thirteen service users with serious mental illness (Nine inpatient, four community-based) participated in semi-structured 1–1 interviews exploring weight management support experiences. Transcript data was analysed using thematic analysis.

**Results:**

Five key themes were developed: service users' experience of weight management conversations, developing therapeutic relationships, support for physical activity and weight management, deliverer characteristic preferences, and user descriptions of MECC.

**Conclusions:**

Service users reported a lack of information about medication-related weight gain and suggested further staff training to improve therapeutic relationships and weight management support for service users with serious mental illness.

**Innovation:**

This study uniquely explores service users' perspectives on weight management conversations within mental health care, applying MECC in a novel context. It highlights the perspective of individuals with serious mental illness on weight-related issues, challenging existing practices, and proposing strategies for integrating physical health support in mental health settings.

## Highlights

• Service users lacked information of medication-related weight gain
• Strong therapeutic relationships encourage engagement in weight management discussions
• Service users value timely, tailored advice and suggest further staff training to enhance weight-related conversations.

## 1 Introduction

Previous research has shown that healthcare professionals (HCPs) find weight management conversations with service users challenging particularly if service users lacked willpower to engage in healthy lifestyle behaviours or had lack of understanding of the health benefits of weight management [1]. Michie found HCPs often avoid discussing weight management unless medically necessary, fearing emotional reactions from service users [2].

Weight management conversations are crucial for service users with serious mental illness (SMI), as they face increased obesity prevalence, often worsened by antipsychotic medication use [3]. In England, adults with SMI are nearly twice as likely to be living with obesity compared to the general population [4]. Weight management is particularly challenging for these individuals due to factors such as inadequate physical activity, especially in inpatient settings [5], the metabolic effects of antipsychotics [6], poor diet, and the use of food as a coping mechanism for low mood or depression [7]. Recent research highlights the potential for effective weight management even in those taking psychiatric medications. Wharton et al. [8] found that participants in a weight management programme achieved significant weight loss regardless of antidepressant or antipsychotic use. Similarly, Imayama et al. [9] showed that antidepressant use did not hinder adherence to a diet and exercise programme or improvements in metabolic health. These findings underscore the importance of tailored interventions that address both mental and physical health needs of people living with SMI.

A public health initiative which focuses on delivering opportunistic health-related behaviour change conversations is Making Every Contact Count (MECC) (See Supplementary file 1, TiDIER table for further context of MECC in the mental health organisation). MECC is described as a ‘person centred and opportunistic approach to health behaviour change…through conversations and topics around smoking, physical activity, healthy diet and alcohol’ [10]. Although HCPs perceive a service-user benefit of MECC, engagement with MECC conversations is low [11,12]. Recently, in a mental health inpatient setting in Northern England, a training package was developed to enhance staff confidence and ensure consistent delivery of the MECC approach across this setting. The package employs a ‘train the trainer’ model, enabling peer-to-peer training [13]. Additionally, a tailored version of MECC was created, integrating the *A Weight Off Your Mind* programme, a regional weight management plan for individuals with learning disabilities or severe mental illness focussing on physical activity and diet to help service users achieve a healthy weight through MECC conversations [14]. A fidelity assessment of this MECC approach demonstrated moderate fidelity to the MECC guidance and high fidelity in the delivery of the training package, suggesting its effective implementation and potential for public health benefits [15].

Research on service user perspectives shows receptiveness to well-timed behaviour change conversations from HCPs [16]. While some report inconsistent weight management information [17], others emphasise the importance of positive HCP relationships with ongoing care for successful conversations [16]. Koball et al. [18] found HCP's weight loss recommendations motivated service users to adopt healthy behaviours. Research shows HCPs find opportunistic weight management conversations motivate service users' weight loss [19] and service users are receptive to such advice [20]. However, studies on service users' perspectives of these conversations are lacking. This study explored service users' perspectives on weight management conversations within a Northeast England healthcare organisation, focusing on individuals with SMI (both inpatient and community-based). Specifically, it examined how these conversations were experienced by service users, the factors that influenced their outcomes, and the role of therapeutic relationships with HCPs in facilitating or hindering these discussions.

## 2 Material and methods

### 2.1 Design

This study adopted a social constructionist/relativist, descriptive approach, employing qualitative, semi-structured one-to-one remote interviews, exploring service users' experiences of weight management conversations and forming relationships with HCPs. Participants were current inpatients or community-based individuals with current or previous SMI treatment history. The protocol was published elsewhere [21] preregistered and can be found on the Open Science Framework: https://osf.io/ewktc [22].

### 2.2 Participants and setting

The setting for this research took place in Northeast England in a healthcare organisation managing multiple mental health services across the region.

Purposive sampling was used to recruit service users across inpatient and community-based settings, ensuring appropriate representation of age and gender. The research team liaised with a member of staff from the healthcare organisation (Author, SF) who passed on details of the study (participant information sheet) to ward managers. Inpatient service users were approached by members of staff they were familiar with and invited to an interview. Inclusion criteria for participation included being aged 18+ and either currently or previously having been a service user with SMI of the healthcare organisation.

### 2.3 Procedure

Interviews were arranged via Microsoft Teams through a central staff member to ensure no personal details from the service users, such as email addresses, were shared. Participants that were currently in hospital had a chaperone assist them during the interview. Community based service users were recruited through an involvement service which was set up internally through a member of staff from the healthcare organisation (Author, KM). All interested community-based participants provided their email addresses to be contacted by the lead author (EK) who arranged and conducted all interviews. EK (PhD) is a female research associate and qualitative researcher with experience in conducting interviews with participants and using NVivo software to analyse qualitative data. Participants were not known to the interviewer prior to interviews commencing and were informed that the interviews were for research purposes. All participants read the participant information sheet and provided either verbal or written consent. Interviews, lasting 15 to 60 min, were audio recorded and transcribed verbatim using transcription services, for analysis. Data collection was informed by information power [23] and continued until authors felt enough information was captured by the interviews to meet the aims of the study. Transcripts were checked for accuracy and anonymised by author EK.

### 2.4 Materials

A topic guide (Supplementary file 1) was adapted from Rodrigues et.al [24] who based the service user questions on previous participant interactions. The topic guide covered beliefs about mental health and diagnosis, experiences of communication about weight management, training/resource need and health professional characteristic. Alongside the topic guide, service users were also shown examples of MECC conversation starters, for example ‘How important is it for you to eat healthily?’ and ‘What physical activities do you enjoy doing?’ (Supplementary file 1) (provided to the research team through the healthcare organisation, Authors SF and CR) relating to weight management, physical activity and alcohol consumption as shown to staff during MECC training sessions.

### 2.5 Data analysis

All transcripts were coded in NVivo using reflexive thematic analysis [25] The first stage of analysis involved authors familiarising themselves with the data, author EK read through all transcripts and made initial notes on the transcript of anything that appeared relevant to the research question. All transcripts were then initially coded by author EK who coded all relevant sections which could help to address the research question. To ensure a rigorous analytic process, author AR independently coded 10 % of the transcripts and met with EK to discuss initial codes and any discrepancies. Once coding agreement was reached, EK conducted the remaining analysis. Authors AR and EK then discussed potential themes by grouping the codes. Final themes were firstly reviewed between authors AR and EK and then by all authors to ensure analytic agreement. Naming of themes was agreed between authors before the final write-up of the analysis.

### 2.6 Ethical statement

Ethical approval for this study was granted from Northumbria University Research Ethics Committee (Ref: 43190). All participants provided informed consent.

## 3 Results

### 3.1 Participant characteristics

Thirteen service users took part in 1–1 interviews including nine in patients receiving mental health treatment and four community service users of which two were currently receiving mental health treatment. Participant characteristics are presented in Table 1.

**Table 1:** Characteristics of service users.⁎

Participant	Gender	Inpatient/Community	Receiving mental health support	Medication
Participant 1	Male	Inpatient	Yes	Antipsychotic
Participant 2	Female	Community	No (GP care for weight management)	Antidepressant
Participant 3	Male	Inpatient	Yes	Antipsychotic
Participant 4	Male	Inpatient	Yes	Antipsychotic
Participant 5	Male	Inpatient	Yes	No medication
Participant 6	Male	Inpatient	Yes	Yes (not specified)
Participant 7	Male	Inpatient	Yes	Yes (not specified)
Participant 8	Male	Inpatient	Yes	Antipsychotic
Participant 9	Male	Inpatient	Yes	No medication
Participant 10	Transgender female	Community	Yes	Yes (not known)
Participant 11	Male	Community	No (outpatient services for brain injury)	Not disclosed
Participant 12	Transgender male	Community	Yes	Yes (anxiety)
Participant 13	Male	Inpatient	Yes	No medication

⁎ Service users were given the opportunity to provide demographic information. Ethnicity and age were not disclosed.

Overall, five overarching themes emerged from the analysis, which align with the study's aim of exploring service users' experiences of weight management conversations and their relationships with HCPs. These themes cover service users' perspectives on: (1) their experiences of weight management conversations, (2) the development of therapeutic relationships in hospital settings and how these shifted after discharge for community service users, (3) the support they received from HCPs, (4) their preferences for HCP communication, and (5) their descriptions of MECC conversations. To illustrate the key themes identified in the analysis, direct quotes from participants are presented throughout the results section. These quotes provide contextual depth and highlight the perspectives and experiences of participants.

### 3.2 Theme 1: Experience of weight management conversations

#### 3.2.1 Fear of judgement from HCPs

Service users discussed their experiences of weight management conversations they had with staff during inpatient stays and receiving community care. There appeared to be judgement around weight management conversations, which impacted engagement, due to feeling too embarrassed to discuss dietary behaviour. Participant 2 discussed feeling how community-based HCPs could be ‘quite judgemental’ when discussing weight. Although participants felt like a direct approach was needed when having weight management conversations to initiate behaviour change, participant 2 spoke of feeling judgement from HCPs and preferred HCPs to be more understanding of individual situations:

*‘You don't want to feel as if you're being judged… I mean I understand that obviously there are situations where they do have to be straight to the point and frank about it.’ (Participant 2, Community).*

Inpatient service users discussed feeling less open to discussing weight and eating behaviour with HCPs and instead preferring an approach where the HCPs would portion control to assist with weight management. Participants spoke of feeling ‘embarrassed’ to discuss diet particularly if they had experienced recent weight gain.

#### 3.2.2 Weight gain as a side effect of medication

Many service users discussed how medication, particularly antipsychotics had a weight gain side effect. Participant 12 spoke of relying on leaflets to find out information about the medication they were taking as this had not been explained to them at the clinic where they received the medication:

*‘Actually, I think just for general medication as well. I think every time I've ever been given any medication no one's ever said ‘but you might experience this' you have to go back and read the leaflet and be like oh I hope I don't get any of this.’ (Participant 12, Community).*

A sense of acceptance was seen among certain service users that they had no control over the weight gain side effect of the medication as other medications might be less successful in treating their mental health symptoms. They therefore had to accept the weight gain side effect to control symptoms caused by their mental health condition.

#### 3.2.3 Referral to dietitian to support healthy weight management

To assist with weight management, service users mentioned being referred to a dietitian. To promote healthy eating behaviours on hospital wards, service users were referred to dietitians and exercise staff. Dietitians work with service users to encourage them to make their own nutritious meals to maintain a healthy weight whilst staying in hospital. Service users generally had a positive view and experience with the dietitian, a community-based service user mentioned how a referral to the dietitian led to positive behaviour change in making healthier eating choices. When experiencing lack of eating, conversations and information provided by the dietitian helped this service user resume a healthy eating pattern:

*‘…a dietitian gave me a really good guide for returning to eat again following not eating for a really long time.’ (Participant 10, Community).*

#### 3.2.4 Encouragement to engage in physical activity in hospital

Physical activity was often mentioned by service users especially those who were currently inpatients of a mental health hospital. Participant 5 discussed how engaged they were in physical activity through access to the gym and encouragement from the hospital gym staff:

*“Some of the duty staff as well, they do encourage people to go to the gym as well. I've seen a few lads who are quite sedentary, they normally try and encourage them and say, ‘Oh, are coming this time?’” (Participant 5, Inpatient).*

Service users who were keen on participating in physical activity in the hospital gym spoke of having a trusting relationship with gym staff as they were aware of staff being trained for this role, so the service users felt comfortable in the environment with those particular staff. In addition to being encouraged in the hospital gym, it was clear that service users had positive engagements with gym staff whilst on the wards. Service users discussed how often gym staff would be seen to motivate service users to engage with the gym by initiating conversations and maintaining a polite approach to encourage service users to think about keeping themselves fit whilst in hospital.

### 3.3 Theme 2: developing therapeutic relationships with HCPs

#### 3.3.1 Positive impact of building relationships with HCPs for inpatients

Building relationships with HCPs whilst in hospital appeared to be beneficial to the service users so that they could gain useful advice and signposting to information. For Participant 4, HCPs could help them manage expectations of food availability on the ward to encourage healthy weight management:

*‘They basically put things in perspective for us and allow me to see the bigger picture and understand where I'm at. I'm in hospital at the end of the day and you can't have access to all the food that you'd want on the outside. I think they are reaffirming that positive mindset with it as well.’ (Participant 4, Inpatient).*

Some inpatient service users discussed how building good relationships with staff could be positively reinforcing, particularly when having somebody to talk to when engaging with certain activities in hospital such as attending the gym. Consistent engagement with staff on the wards for inpatient service users was a useful way to build positive relationships. A community-based service user spoke of the need to have built a good relationship before engaging with conversations relating to weight management. It appeared to be important for service users to develop trust in HCPs before discussing personal issues, however once a relationship had been built, they felt comfortable to engage in sensitive conversations.

#### 3.3.2 Less contact with HCPs for community patients

Community based service users appeared to have different experiences with building relationships with HCPs as they had less contact than those residing in hospital wards. Service users spoke of how check-ups were lacking and proposed that annual checks be carried out for those with mental health conditions who had been discharged from hospital settings. However, it was unclear if these check-ups were specific to weight management as Participant 12 recalls:

*‘But also, if they just had a random check-up or something every year. You know, because I know some people get an annual check-up at the GP. It would be nice if there was a mental health version.’ (Participant 12, Community).*

Community based service users also mentioned how they would prefer regular contact initiated by HCPs, as they appeared to have a reluctance and lack of confidence to initiate contact themselves. When service users were struggling with their mental health symptoms, they mentioned a need for check-ins from HCPs to assist them.

#### 3.3.3 Tailored advice to improve communication between patients and HCPs

Although service users generally described having positive relationships with HCPs which had beneficial outcomes, there were some service users who highlighted the importance of tailoring the advice being given to them. Service users acknowledged the personal nature of others diagnosed with mental health condition and felt that HCPs needed to be aware of external influences, particularly when discussing the topic of weight management. To improve communication and ensure tailored advice is being provided to individuals, participant 2 highlighted a need for further staff training.

*‘Everyone's different and everyone has their own set of circumstances and own set of external factors as to why they may be overweight, so it has to be tailored to each individual user.’ (Participant 2, Community).*

### 3.4 Theme 3: deliverer of weight management conversations characteristics

#### 3.4.1 Motivating patients to engage in healthy lifestyle behaviours

For HCPs who deliver MECC style conversations, service users mentioned specific characteristic preferences that would assist them to engage in the conversation. Being motivated by HCPs was a key factor in encouraging service users to engage in healthy lifestyle behaviours. One participant spoke specifically about weight management and realised that although healthy weight management was a personal goal, it was easier to achieve with motivation from HCPs. Participant 9 echoed how persistent staff were with motivating service users to partake in healthier lifestyle choices.

*‘They're always wanting to motivate you, so that's a good thing.’ (Participant 9, Inpatient).*

This motivation fed into one-to-one sessions with HCPs where service users spoke of the encouragement and motivation they would receive during these sessions to help them achieve the personal goals they had set.

#### 3.4.2 Skill of knowing how to have MECC conversations with patients

Participant 2 described having an ‘air of tact’ to ensure that conversations were person-centred and tailored to individuals and this was important to several service users.

‘*So, I feel like there has to be an air of tact about it. And how to approach it and how to deal with it obviously varies from person to person.’ (Participant 2, Community).*

As service users had been diagnosed with mental health conditions, they discussed the need for a sensitive approach to be taken when having conversations related to lifestyle factors such as healthy weight management to avoid triggering adverse mental health outcomes. Maintaining a friendly approach helped service users to feel at ease and be more willing to engage with healthy lifestyle conversations with HCPs. Other service users highlighted a preference for a more direct approach to ensure information was being provided and received effectively, however they still maintained a preference for a friendly attitude from HCPs to encourage engagement within the conversations.

#### 3.4.3 Knowing the appropriate time for MECC conversations

Service users raised that to initiate MECC conversations, there needed to be appropriate timing to ensure they would be receptive to it. When discussing weight management, service users reported that HCPs needed to be aware of individual situations of service users before raising the topic, for example if they were struggling with other mental health symptoms, they would be less likely to engage with a weight management conversation. Participant 2 also mentioned that when initiating MECC conversations HCPs should be aware of the sensitive nature of mental health conditions to ensure that MECC focused conversations were raised once the service users feel comfortable engaging with HCPs about specific healthy lifestyle behaviours.

*‘if you're incorporating mental health into it you don't want to just go bowling in straight away with your first meeting with somebody.’ (Participant 2, Community).*

### 3.5 Theme 4: MECC: how service users describe MECC

#### 3.5.1 MECC as brief advice

When discussing MECC, one community-based participant who had experience and knowledge of MECC, described MECC as brief advice and something that when weaved into conversations should be kept concise. This was not reiterated by other participants and no other participants provided views on appropriate length of MECC conversations.

*‘Short and concise and to the point. The difficulty with mental health and MECC is how to make that short, concise and to the point that will register for that person.’ (Participant 10, Community).*

#### 3.5.2 Recalling MECC conversations from examples of MECC conversation starters

During interviews, service users were shown examples of MECC conversation starters (Supplementary file 1). After seeing examples of MECC conversation starters, service users recalled having these conversations with staff. Participant 8 remembered having MECC conversations about weight management on a hospital ward.

*‘With one lass I've talked about my weight with her, and I've done all them chats that are on the page now as well.’ (Participant 8, Inpatient).*

Another participant recalled a MECC conversation on diet and healthy weight management. Staff would provide verbal advice about nutritional information on specific food and drinks to help to promote service users to engage in healthier lifestyle choices such as sugar reduction.

#### 3.5.3 Service users unable to recall MECC conversations

Some service users clearly remembered having MECC conversations with HCPs however, several did not recall MECC conversations taking place even when shown examples of conversation starters relating to core elements of MECC. Participant 5 discussed having group sessions focused on healthy dietary choices and being provided with opportunities to cook healthy food while in hospital, however specific MECC conversations were not recalled.

*‘I personally haven't had any kind of conversation. Staff have mentioned in groups about particular portion sizes with food. More so when we do things like fakeaway when someone cooks a meal, one of the patients cooks a meal for the other patients. Or when there is some baking going on.’ (Participant 5, Inpatient).*

After viewing the examples of MECC conversation starters, both community and inpatient service users discussed not recalling conversations initiated by HCPs in the mental health setting.

## 4 Discussion and conclusion

### 4.1 Discussion

This study reports service user experiences of weight management conversation with HCPs in inpatient and community settings across a Northeast England healthcare organisation. The findings indicate that service users have mixed experiences with weight management conversations. Some reported a fear of judgement from HCPs and expressed a sense of lost control over their weight, particularly when prescribed antipsychotic medication, where weight gain was frequently discussed as a side effect. Despite these concerns, service users valued referrals to dietitians and appreciated hospital staff encouraging physical activity. The quality of therapeutic relationships with HCPs played a crucial role in engagement, with service users more receptive to weight management discussions when a trusting relationship had been established. A friendly yet direct approach from HCPs was preferred, though community-based service users, who had less contact with HCPs, found it harder to build such relationships. Tailored advice was seen as a key motivator for participation in weight management conversations. When HCPs initiated these discussions effectively, service users felt encouraged to adopt healthier lifestyle behaviours. However, service users emphasised the importance of HCPs having the necessary skills and choosing the right moment to introduce the topic to maximize engagement. Awareness of MECC varied. While those familiar with the term associated it with brief advice, recollections of MECC-style conversations with HCPs were inconsistent when presented with specific examples.

Service users shared their experiences of weight management conversations with HCPs, highlighting the need for more direct discussions about medication-related weight gain. While they engaged with dietitian advice, many expressed a desire for clearer, proactive communication from HCPs about potential side effects. This aligns with prior research, which found that individuals with SMI are often insufficiently informed about the side effects of antipsychotic medications [26]. These findings reflect broader issues in patient-provider communication, particularly regarding the role of HCP support in managing weight-related concerns. Previous studies have shown that patients value general practitioners' input but often feel they lack essential information about medication impacts [27]. Moreover, gaps in shared decision-making, especially concerning side effects, remain a persistent issue [28]. For individuals with SMI, weight management conversations often occur reactively, only after significant weight gain has been observed, rather than proactively addressing potential risks early on [29]. These findings must be considered in the context of weight-related stigma in healthcare. A recent multinational study of nearly 14,000 adults revealed that stigma from healthcare providers is widespread, with internalised weight bias linked to avoidance of medical care [30]. This presents a significant challenge; although patients require more support in managing weight changes, the presence of weight stigma may deter them from seeking this help. Addressing this issue requires targeted strategies [30], such as training healthcare providers to recognise and counteract weight stigma, fostering respectful communication that avoids assumptions, and equipping providers with tools to engage in supportive, stigma-free discussions about weight and health. One potential resource to assist in this process could be a virtual patient tool, currently being assessed for feasibility, which could help train providers in having stigma-free conversations [31].

Research has shown that, from a HCP perspective, previous relationships do not need to be built prior to having healthy conversations [32], however findings from this study have shown that for service users with SMI, therapeutic relationships are important, particularly when the conversation is of a sensitive nature. Studies have highlighted the importance of therapeutic listening, responding to patient emotions, and ensuring patient-centred care in fostering these relationships, which are key to improving health outcomes [33]. Parchment et al. [34] found that HCPs perceive service users in receipt of MECC unwilling to take part in healthy conversations, which they see as a barrier to engagement. Service user perspectives from our study indicate that the characteristics of HCPs and how they deliver conversations are crucial in encouraging engagement. For example, being ‘tactful’ and ensuring advice is tailored to individuals were highly valued by our participants. This aligns with findings that SMI patients expect mental health nurses to provide support by personalising their care and identifying their needs, which strengthens the therapeutic relationship and aids recovery [35].

Currently in the healthcare organisation, MECC training is provided to staff, however the findings from our study show how HCPs could benefit from additional, targeted training on specific components, such as providing advice to service users regarding medication and weight gain side effects and ensuring that HCPs offer tailored advice at appropriate times to encourage engagement and promote healthy behaviour change. In particular, training in motivational interviewing (MI) could enhance HCPs' ability to engage service users more effectively [36]. MI is a technique used to motivate service users to make healthy behaviour changes through a partnership of communication, empathy, and compassion, which is essential in fostering a strong therapeutic relationship. Furthermore, integrating health coaching principles, such as personalised self-management plans and collaborative goal setting, could complement MI and support HCPs in empowering service users [37,38]. Including communication tools such as MI and health coaching could further equip HCPs to engage with vulnerable SMI populations. These skills are crucial for maintaining a therapeutic relationship that promotes trust and a sense of partnership in the health improvement process. The integration of therapeutic communication strategies, such as MI, into routine practice would help ensure HCPs can better connect with service users, facilitating more meaningful and productive health conversations.

While service users in our study may not explicitly recall MECC conversations, they did remember discussions around health behaviours such as diet and exercise. This aligns with a recent consensus study [10] that the distinguishing feature of MECC is the approach and mechanisms applied to conversations, rather than their explicit labelling. To operationalise MECC more effectively with service users, future training could focus on refining the conversational mechanisms that support health behaviour change, drawing on the operational definition [10]. Additionally, training could further emphasise a MI approach that is opportunistic, person-centred, and focused on boosting users' self-confidence and supporting individuals identifying their own solutions [10].

To translate these insights into practical service improvements, we can draw on behaviour change approaches, such as the Behaviour Change Wheel [39] to identify specific implementation priorities. Targeting MECC training specifically toward prescribing professionals addresses a crucial gap [training; psychological capability, physical opportunity], particularly for supporting weight management conversations with patients experiencing severe mental illness, where medication-related weight gain presents unique challenges. The implementation of digital support tools, such as the MECC Gateway App, can extend support beyond the initial hospital admission [enablement; physical opportunity], and can support healthcare professionals in providing consistent support beyond the initial hospital admission. Resources such as this have been shown to facilitate MECC implementation in other settings [40]. This digital infrastructure, combined with a virtual patient tool that allows healthcare professionals to practice sensitive conversations in a safe environment [modelling; social opportunity], creates a comprehensive approach to service improvement. Importantly, our findings emphasise the value of incorporating service user perspectives in program development [enablement; social opportunity]. This participatory approach has enhanced our understanding of what constitutes helpful conversations and effective support mechanisms, allowing for more tailored and responsive service delivery. As Harrison et al. [41] highlights, such initiatives need to consider both wider support networks and long-term sustainability beyond initial interventions, suggesting the need for a co-productive approach that addresses both individual and community-level wellbeing.

This study has contributed to the literature through providing a service user perspective of weight management conversation which is currently lacking however, there are limitations to consider. Although, male, female, and transgender participants took part, the sample in this study was predominantly male, therefore conclusions cannot be generalised to other genders who may have different experiences with weight management conversations. This study did not account for cultural differences among service users or collect data on participants' ethnicity, limiting the ability to assess cultural implications. Future research should explore the role of culture in weight management conversations within mental health settings, particularly how different cultural backgrounds and dietary norms shape perceptions of body weight and diet. Additionally, perspectives from individuals of diverse genders should be considered to ensure a more inclusive understanding of these discussions. This study was also limited to one Northeast England based mental health setting therefore cannot reflect what might occur in other mental health settings. Future research showing how service users perceive weight management conversations from HCPs in other geographical settings would help to support the findings from this study.

### 4.2 Innovation

By applying the MECC approach in a mental health context, this study introduces a novel application, typically used in general healthcare, to a specialised and vulnerable population.

Based on the findings from this study, we make recommendations for improving the service user experience whilst receiving mental health treatment in either hospital or community settings. The findings have highlighted the importance of weight management conversations between HCP and service users, with a particular focus on discussions around the potential weight gain side effect of medication. Our recommendations focus on targeting conversations to provide service users with appropriate advice, enabling informed decisions and better weight management treatment whilst receiving mental health services.

### 4.3 Conclusion

This study reports novel findings outlining service user experience of weight management conversations in a hospital or community based mental health setting. We highlight the factors that encourage service user engagement with weight management conversations for example, importance of HCPs conversation skills, motivation from HCPs and providing tailored weight management advice to individuals. MECC has shown to be a useful method to utilise opportunities to provide brief advice, however service users have described that such conversations need to occur at appropriate times to maximize engagement.

## Availability of data and materials

The datasets used and/or analysed during the current study are available from the corresponding author on reasonable request.

## CRediT authorship contribution statement

**Emma Kemp:** Writing – review & editing, Writing – original draft, Project administration, Formal analysis, Data curation. **Catherine Haighton:** Writing – review & editing, Funding acquisition, Conceptualization. **Sally Faulkner:** Writing – review & editing, Validation. **Kate McBride:** Writing – review & editing, Validation, Funding acquisition. **Maria Raisa Jessica Aquino:** Writing – review & editing, Funding acquisition, Conceptualization. **Rob Wilson:** Writing – review & editing, Funding acquisition, Conceptualization. **Milica Vasiljevic:** Writing – review & editing, Funding acquisition, Conceptualization. **Craig Robson:** Writing – review & editing, Validation, Resources. **Mish Loraine:** Writing – review & editing, Validation, Funding acquisition, Conceptualization. **Jill Harland:** Writing – review & editing, Validation, Resources. **Angela M. Rodrigues:** Writing – review & editing, Writing – original draft, Supervision, Project administration, Methodology, Investigation, Funding acquisition, Formal analysis, Conceptualization.

## Funding sources

This study/project is funded by the National Institute for Health and Care Research (NIHR) Applied Research Collaboration (ARC) North East and North Cumbria (NIHR200173). The views expressed are those of the author(s) and not necessarily those of the NIHR or the Department of Health and Social Care.

## Declaration of competing interest

The authors declare that they have no known competing financial interests or personal relationships that could have appeared to influence the work reported in this paper.. KM and SF are the employed by the healthcare organisation where the study took place, and supported this project in terms of recruitment and dissemination.

## Appendix A Supplementary data

Supplementary material Image 1
