# Supplementary material for: A qualitative exploration of service users' experiences of weight management conversations in a mental health setting
Source: PEC Innov. 2025 Mar 22;6:100389. doi: 10.1016/j.pecinn.2025.100389 (PMC11992401; doi:10.1016/j.pecinn.2025.100389)
Supplement: Supplementary file 1 — Supplementary material [file mmc1.docx]

1. TIDieR Checklist- MECC implementation at Northeast England healthcare organisation

**Table 1. TIDieR for the implementation of MECC across the healthcare organisation**

| TIDieR Checklist Item | Mental Health Setting MECC implementation |
| --- | --- |
| Name | Making Every Contact Count (MECC) implementation |
| Why  (Identified from documents:  MECC logic model,  MECC fact sheet,  MECC evaluation framework,  MECC plan  Implementation checklist,  MECC implementation guide) | The primary aim of the MECC training programme in the trust is to:   - Improve staff confidence in having opportunistic healthy lifestyle conversations with service users. - To deliver healthy lifestyle messages and encourage service users to change their behaviour. - Supporting service users to make positive changes to their physical and mental health. - Increase number of MECC conversations that staff are having with service users about healthy weight, physical activity and healthy eating. - To highlight considerations for the evaluation of MECC programmes that are specific to mental health settings. |
| What | Materials: Pre- and post-training questionnaires were sent to all participants of the training to enable internal training evaluation.  All training resources and copies of training slides are added to the MECC hub website.  Procedure: A training package was designed by the trust to deliver two training pathways. The first was a ‘train the trainer’ session to enable staff to gain the skills needed to deliver cascade MECC training to their colleagues. The second was a bespoke training which combined Core MECC training with the element of A Weight Off Your Mind (AWOYM) to provide staff with the skills and information needed to deliver MECC conversations. Prior to September 2022 Core MECC training was delivered without the addition of AWOYM. Training was delivered between both in online and face-to-face formats.  Planning and implementation of training through AWOYM steering group. |
| Who | The training is designed by the public health and wellbeing lead in the trust. Train the trainer training is delivered by a wellbeing specialist/regional MECC trainer within the trust. Bespoke training is delivered by a health improvement specialist with the trust. Clinical staff working in mental health settings across the trust are invited to take part in the training. |
| How | Online training via Microsoft Teams  Face-to-face within hospital settings |
| Where | Northeast England mental health setting |
| When and how much | Post COVID-19, the training package relaunched in September 2022. The initial planned sessions occurred between September-December 2022, then recommenced in March 2023 and are currently ongoing.  Train the trainer training sessions lasted 3 hours, and Core MECC + AWOYM were 90 minutes. 5 train the trainer and 15 core MECC training sessions have been delivered since September 2022. |
| Tailoring | Both training sessions included showing staff examples of MECC scripts for AWOYM following the 3A’s structure which focused on healthy weight management, physical activity and alcohol consumption to prepare staff how to deliver MECC conversations. |
| Modifications | No modifications |
| How well | No intervention adherence or fidelity assessed |

2.Service user interview topic guide

**MECC Service user interview topic guide**

Thank you for agreeing to take part in this interview. We are inviting service users so I am pleased you are able to share your views with us. We are interested in your feedback on the MECC approach at CNTW.

| **MECC approach at CNTW**  *Remind participants of the definition of MECC: “Making Every Contact Count (MECC) is an approach to behaviour change that uses the millions of day-to-day interactions that organisations and people have with other people to support them in making positive changes to their physical and mental health and wellbeing. The MECC approach at CNTW focus on weight management advice.”* |
| --- |
| **Beliefs about mental health diagnosis and weight** |
| How long you have been suffering from [diagnosis]?(probe for: pre and post diagnosis)  What is your understanding about what [diagnosis] is?  What treatment/s have you had/do you know what is available?  What do you think the link is, if at all, between weight and [diagnosis and/or medication]? |
| **Experiences of communication about weight management** |
| How do you feel about communicating with health professionals about weight management? (Probe: easy, comfortable, difficult, who initiates the dialogue between health professional and patient)?  If a health professional (for example a doctor, nurse, AHP, exercise therapist, support staff, pharmacy, etc) from CNTW has introduced the topic of weight management in a conversation can you remember how they did this?  What did they say? Can you give me an example?  Are there times when weight hasn’t been discussed, if so, when? (probe for possible barriers)  How do you feel when health professionals start talking about weight management within a consultation  Did the health professionals that raised the topic of weight management seem at ease? (can you give me an example)  How did you feel when health professionals were discussing the topic of weight management with you? (uncomfortable/comfortable)?  When health professionals are talking to you about weight management how do you prefer them to be (probe for: direct, friendly, empathic, sensitive, what type of language do you prefer them to use: obese, overweight, BMI).  How would you sum up your experiences of communication with health professionals in general? |
| **Training/resource needs** |
| Reflecting now after your conversation is there anything that they (health professionals) could have done/said differently?  Would health professionals benefit from more specialised training when talking about weight management?  What do you think could be done to improve communication between health professionals and people living with serious mental illness regarding weight management?  How do you feel about the information/resources/support available to you to help with weight management? |
| **Specific health professional characteristics** |
| How might a health professional help you lose weight, why do you think it worked? (Probe for empathy/understanding, rapport building, communication skills, set specific goals, motivated)  Do you think if a health professional had spoken to you about weight management (many years ago) it would have made a difference (for example given you the confidence to lose weight)?  Are there any specific qualities (probe for: communication skills, empathy) that a health professional displays that may make you more likely to listen to/ be motivated by them to change when discussing weight management?  Looking back, when would be the best time a health professional could raise the topic of weight management? (probe for: before particular medication was prescribed (antipsychotics); during a consultation about an unrelated health issue, linking to a comorbid condition such as diabetes, hypertension, routine preventive check up).  Is there anything that I haven’t asked you that you would like to add or say?  Close by thanking them very much for their time and ask if there is anything they would like to ask. |
| **Ending the interview:**  Clarify and summarise main points.  Is there anything else you’d like to say?  Close the interview and thank the interviewee for their participation. |

3.Examples of MECC conversation starters

 Example of MECC conversations for Healthy weight management:​

- Ask- **How important is it for you to eat healthily?**​
- Assist- **Would you like to improve what you eat?**​
- Act- **What types of drinks do you have in a typical day?**

Physical Activity

- Ask- **Do you know the benefits physical activity has on your health and wellbeing?**
- Assist- **You have been told you are inactive/sedentary do you know what this means?**
- Act- **What physical activities do you enjoy doing?**

Alcohol

- Ask- Do you mind if I ask a question about Alcohol?
- Assist- Would you like any support to help you drink less?
- Act- How about trying a few alcohol free nights a week?

4.Table of themes and quotes from analysis

Table 2: Themes and descriptions from thematic analysis

| Themes | Sub-themes | | Description | Quotes |
| --- | --- | --- | --- | --- |
| Experience of weight management conversations | | - Weight stigma - Weight gain as a side effect of medication - Referral to dietician | Theme 1 describes how service users expressed experiences of having weight management conversations with HCPs. This included a stigma around weight, being uninformed of the weight gain side effect of antipsychotic medication and referrals to dietician to assist with weight management. | ***Weight stigma****:*  *‘Because there’s some, like some health professionals I find that can be quite, you feel as if they’re being quite judgemental towards you about it.’ (Participant 2, Community)*  *‘Or if I eat too much. If I’m eating too much then I prefer them just to give me less food instead of saying anything to me. I might get a bit embarrassed.’ (Participant 7, Inpatient)*  ***Weight gain as a side effect of medication****:*  *‘Yeah, absolutely. Yeah. I mean especially when you start going on Google and trying to find out things there. You’re just like I wish someone had just explained it to me at the clinic.’ (Participant 12, Community)*  *‘There is nothing I can do about it. it’s the medication that’s putting on weight. I haven’t thought about things like this because it’s, like, “Oh you can do this and you can do that.” It just won’t work. I’m on Clozapine. Every single person on this ward that I’m on has got a weight problem because of the medication.’ (Participant 3, Inpatient)*  ***Referral to dietitian:***  *‘It’s been brought up on the ward a couple of times because I’ve actively said, “Listen, I don’t eat the food that they serve here. Is there any way I can try and make it so I can cater for myself a bit easier?” That’s when I got referred to the dietician and that and referred through the gym and stuff like that.’ (Participant 1, Inpatient)* |
| Developing therapeutic relationships with HCPs | | - In-patient relationships - Community contact with HCPs - Tailored advice to improve communication | Theme 2 underscores the valuable therapeutic impact of the relationships formed between staff and in-patients. There is a clear desire among community service users for this connection to persist beyond their in-patient experience. There is also an identified need for staff to offer more personalised guidance to individuals. This could be effectively accomplished through additional training opportunities for staff members. | ***In-patient relationships:***  *‘It doesn’t matter who is giving you the information, or where you are getting it from. No, not really any different. Not really. I feel like I can talk to all of them about it and as I say get good advice from them each time, yes.’ (Participant 4, Inpatient)*  *‘But there are other times where we’ll have a good rapport, where we’ll have good, long conversations. It’s a mix. It’s a combination of everything. It’s good rapport, reinforcements and also allowing me to just get on and train, rather than just sitting there idly having a conversation for 30 minutes and not doing anything.’ (Participant 4, Inpatient)*  *‘Do you know what I mean? And not to then have, be bombarded with information about losing weight or whatever, like so I would probably say once a relationship has been built up between the service user and the health professional, I think that may be a safer time to kind of bring it in.’ (Participant 2, Community)*  ***Community contact with HCPs:***  *‘Whereas personally I would prefer checks, you know, because there are times where I can’t pick up the phone or I’m struggling, and I just don’t have the confidence to talk to them.’ (Participant 12, Community)*  *‘So yeah, maybe. Maybe there would be some benefit in extra training around that area of like delivering news or talking about it or whatever.’ (Participant 2, Community)*  *‘I think the more knowledge they have in those areas the better advice they can give, and the more accurate advice they can give as well.’(Participant 5, Inpatient)*  ***Tailored advice to improve communication:***  *‘Everyone’s different and everyone has their own set of circumstances and own set of like external factors as to why they may be overweight or whatever, so yeah, it has to be tailored to each individual user.’ (Participant 2, Community)* |
| Support received for MECC components | | - Physical activity - Weight management | Theme 3 relates to service user discussions of two MECC core elements; physical activity and weight management and how interactions with HCPs took place on both components. | ***Physical activity:***  *‘I don’t need motivating. But the three main facilitators here in the gym, I’m not talking about normal staff now, they’re doing their job 100%, in the physical aspect, for a reason. You know? They’re trained for that. So I’m relaxed in that environment, because we’re on the same hymn sheet. You know?’ (Participant 13, Inpatient)*  *‘The gym staff are very polite. Every time they’re on the ward they want to get people engaged in going to the gym, keeping themselves fit. Also, the dietician just keeps your portion control, so then you’re not overweight.’ (Participant 9, Inpatient)*  *‘Well, actually, eight months ago, over a ten-session period, ten weeks, an outside lady came in, and the yoga was a more relaxing type, meditative type yoga. I’m more physical, stressing out, on my type of yoga. But that was a good thing. I did all the sessions. It ended up just one or two of us doing it; most guys, it wasn’t for them’. (Participant 13, Inpatient)*  ***Weight management:***  *‘Yeah, I got a nutritionist to come in to tweak it for me, to see if I could, because there’s one on premises, so I thought, “Right.” And she said, “Well, there’s not really much I can do with you.” You know? “Just make sure you don’t overdo it on the fruit, maybe,” because when I first got here, fruit was free, so I was overdoing it on the fruit, but anyhow.’( Participant 13, Inpatient)*  *‘I wouldn’t say supportive, because everyone is supportive in what they, but the more useful people would be like for diet advice and stuff would be gym lads and stuff like that.’ (Participant 1, Inpatient)* |
| Deliverer characteristics | | - Motivation - Conversation skills | Theme 4 describes the characteristics of deliverers of MECC (HCPs) from service user perspective. Service users discussed feeling motivated by HCPs and defined specific conversation skills that HCPs would display when interacting with them. | ***Motivation:***  *‘I guess the biggest thing is just the encouragement isn’t it? Really it’s up to me to want to lose the weight and try and implement it. But it’s just the encouragement and the advice and that offered I guess.’(Participant 1, Inpatient)*  *‘Yes, doing one-to-one sessions, they do try to focus on the goals you’ve set.’( Participant 5, Inpatient)*  ***Conversation skills***  *‘I feel like there does need to be an air of like friendliness about it and sensitivity about it because obviously if this person is also then suffering with mental health issues like their weight may be a massive trigger. It may be something that sets them back.’ (Participant 2, Community)*  *‘I do like a more direct approach to it. I don’t like beating around the bush as they say. I prefer if they are friendly but they are direct and they say it how it is. Then at least you know for sure what’s going on, what you might need to change.’ (Participant 5, Inpatient)* |
| MECC: User descriptions | | - MECC as brief advice - Recalling MECC conversations - Lack of MECC conversations - Appropriate timing of conversation | Theme 5 highlights how service users described MECC which was mostly brief advice. Service users recalled either having/no having MECC conversations in response to being presented with conversation starters aimed at HCPs to provide prompts for initiating MECC conversations. | ***MECC as brief advice:***  *‘Three minutes max, and even then I’d be tempted to go and say it’s too much for MECC purposes.’( Participant 10, Community)*  ***Recalling MECC conversations:***  *‘I remember a while ago a member of the gym staff had come on a meeting, put a can of orange juice on the table, and we get little sachets of sugar, and put all these sugar on the table, and said “That’s how much sugar is in this one can.” There was a lot.’ (Participant 9, Inpatient)*  ***Lack of MECC conversations:***  ‘So, I was never really like sat down and had like a conversation about any of these.’(Participant 2, Community)  ‘Yes, so I tend not to talk about that kind of thing with staff on the ward. But yes, I’ll talk to other people and that about food and stuff and that.’ (Participant 1, Inpatient)  ***Appropriate timing of conversation:***  *‘I don’t know. That’s a hard one because obviously if you’re incorporating mental health into it you don’t want to just go like bowling in and like straight away with like your first meeting with somebody.’ (Participant 2, Community)* |
